# Capillary zone and agarose plasma protein electrophoresis in the sand tiger shark (*Carcharias taurus*)

**DOI:** 10.3389/fvets.2025.1580744

**Published:** 2025-05-09

**Authors:** Carolyn Cray, Jeny Soto, Michael W. Hyatt, Emily F. Christiansen, Kady Lyons, Jennifer T. Wyffels

**Affiliations:** ^1^Division of Comparative Pathology, Department of Pathology & Laboratory Medicine, University of Miami, Miami, FL, United States; ^2^Wildlife Conservation Society, New York Aquarium, Brooklyn, NY, United States; ^3^NC Aquariums, Raleigh, NC, United States; ^4^Georgia Aquarium, Atlanta, GA, United States; ^5^Marine Science Research Center, Ripley’s Aquariums, Myrtle Beach, SC, United States; ^6^Delaware Biotechnology Institute, University of Delaware, Newark, DE, United States

**Keywords:** acute phase response, *Carcharias taurus*, capillary zone electrophoresis, elasmobranch, lipoproteins, protein electrophoresis, sand tiger shark

## Abstract

Protein electrophoresis is a tool used in the health assessments of non-mammalian vertebrates. In elasmobranchs, agarose gel electrophoresis (AGE) has been described in various species and a newer method called capillary zone electrophoresis (CZE) has been developed and implemented in the undulate skate (*Raja undulata*) and nursehound shark (*Scyliorhinus stellaris*). The study goals were to implement AGE and CZE methods on plasma samples from the sand tiger shark (*Carcharias taurus*) and examine differences in resolution as well as to calculate reference intervals (RI). Plasma was obtained from aquarium sharks (*n* = 23) and free-ranging sharks (*n* = 62) sampled during field research conducted from 2017 to 2023. As with previous reports, CZE was found to provide superior resolution with definition of two major globulin migrating fractions compared to AGE. Overall, the alpha and beta migrating fractions were well correlated between the methods (*r* = 0.92, 0.89, respectively, *p* < 0.0001). The correlation for the gamma fraction was weaker (*r* = 0.42, *p* = 0.002) as the CZE fraction was lower in concentration versus AGE. There were minor, but significant, differences between the concentration of some of the fractions in samples from sharks under managed care versus free-ranging animals which necessitated the production of two sets of RI. In total, this information may help in further studies to address the applicability of these tools in the management of this species under human care as well as in health assessments of free-ranging sharks.

## 1 Introduction

Many shark species are recognized as threatened due to habitat loss and overfishing and remain understudied by researchers and veterinarians. The sand tiger shark (*Carcharias taurus*) is one such species, currently classified as vulnerable globally, and is the focus of many recent studies (1–3). Over the past 20 years, health assessments of free-ranging sharks (*in situ*) have been undertaken with the goal of monitoring these populations and learning more about health and physiology parameters (4–6). At the same time, the sand tiger shark has been a popular species displayed in public aquaria (ex situ) where similar assessments can be undertaken to increase the knowledge base of this species (7). Studies of both groups are valuable to ensure the health and well-being of animals that are maintained under managed care as well as to prioritize health threats and conservation priorities for free-ranging populations.

Protein electrophoresis (EPH) has become a mainstay in routine bloodwork in non-mammalian species providing a relative and accurate quantitation of major serum or plasma proteins including albumin and globulins, and a view of ongoing acute phase responses (8). Overall, EPH has been recognized as an adjunct tool in the detection of clinical and subclinical inflammation and for prognostication (8). This technique has been extensively studied in elasmobranch species. In cownose rays (*Rhinoptera bonasus*), the electrophoretogram from agarose gel electrophoresis (AGE) was dominated by beta globulin migrating fractions in the absence of albumin (9). Cholesterol electrophoresis allowed for the relative quantitation of HDL, VLDL, and LDL fractions and could be correlated with globulin fractions in the protein electrophoretogram (9). Similarly, by AGE methodology, the electrophoretogram of bonnethead (*Sphyrna tiburo*), blacknose (*Carcharhinus acronotus*), white spotted bamboo (*Chiloscyllium plagiosum*), blacktip (*Carcharhinus limbatus*), bull (*Carcharhinus leucas*), lemon (*Negaprion brevirostris*), sandbar (*Carcharhinus plumbeus*), tiger (*Galeocerdo cuvier*), and nurse (*Ginglymostoma cirratum*) sharks were also defined (10–13). In the past several years, human clinical pathology has moved from the use of AGE methods to capillary zone electrophoresis (CZE) which affords an ease of use, increased resolution, and better reproducibility (8). Many veterinary laboratories also now have the capacity to use this method and research reports have characterized the CZE electrophoretogram and provided baseline reference intervals (RI) for the undulate skate (*Scyliorhinus stellaris*) and nursehound shark (*Scyliorhinus stellaris*) (14).

The primary goal of this study was to define and compare AGE and CZE methods in the sand tiger shark. In addition, using the CZE method, free-ranging sharks were compared to those under managed care, and RI were calculated to aid in future health assessments of both populations.

## 2 Materials and methods

### 2.1 Aquarium-based shark samples

Samples were obtained from sharks during routine exams from three aquariums (*n* = 23) between March and November 2021–2024. This was inclusive of 1 juvenile (male), 1 unknown age (female), and 21 adult sharks (8 males, 13 females). These samples represented individual animals (i.e., there were no duplicate analyses). All animals were clinically normal. Blood samples were collected from the caudal vein during routine physical examinations, transferred to a lithium heparin tube, and gently inverted 5–10 times. Plasma was separated by centrifugation, aspirated, aliquoted into cryovials and frozen (−80°C). A vial of frozen plasma was sent overnight with dry ice for plasma analysis. Use of banked samples from sharks housed at Ripley’s Aquariums was approved by the Ripley’s Aquariums Research Review Committee. The period of storage time prior to analysis was variable (approximately 1 week – 3.5 years).

### 2.2 Free-ranging shark samples

Samples were obtained from free-ranging sharks as part of population health studies (*n* = 62) conducted between March and May 2023–2024 and October–November 2017. This was inclusive of 35 adults (13 males, 22 females), 24 juvenile (7 males, 17 females), and 3 subadults (3 males). All animals underwent a physical examination and were found to be apparently healthy. In South Carolina and North Carolina, sharks were sampled as part of ongoing sand tiger shark research surveys using bottom longline gear with all handling protocols approved by Georgia Aquarium’s animal care committee (IACUC # GAI-21-07), Ripley’s Aquariums Research Review Committee, and North Carolina Aquariums Research Review Committee. At capture, sharks were measured, and a blood sample was taken from the caudal vein, placed in a lithium heparin tube and gently inverted, and stored chilled (~4°C) until processing later in the day. Sharks were assessed for any obvious signs of distress (e.g., large bite wounds, emaciation, etc.). At the field lab, blood was centrifuged at 5,000 RPM and plasma was aspirated and stored frozen (−20°C) until transfer to -80°C at the main facility. The period of storage time before analysis was variable (approximately 2 months to 3 years). Samples were shipped on dry ice for laboratory analysis.

### 2.3 Agarose gel electrophoresis

Samples were analyzed using the SPIFE 3000 system (Helena Laboratories, Inc., Beaumont TX, 77707 USA) and split beta gels. Plasma was diluted 1:2 using phosphate buffered saline and analyzed per manufacturer instructions. There was no significant difference present in resolution or fractions between serial dilutions 1:2 through 1:8 (Supplementary Figure 1). The dilution of 1:2 was used based on experience with other elasmobranchs and other species. Gels were scanned and analyzed using SPIFE software. Fraction delimits were placed to obtain percentages using conventions applied from previous elasmobranch publications (see discussion) in tandem with inflection points of consistently observed fractions and were completed by one author (J.S.). Absolute protein (g/dL) values were obtained by multiplying percentages by total plasma protein which was determined by biuret methodology on the Ortho 5600 chemistry analyzer (Ortho Vitros Diagnostics, Rochester, NY, 14626 USA). The mean (of 3 samples run 8 times) intraassay coefficient of variation for the fractions 1 to 4 were as follows: 28.9, 3.0, 1.4, 8.9%.

### 2.4 Capillary zone electrophoresis

Samples were analyzed using the Capillarys 2 Flex Piercing System (Sebia, Norcross, GA 30093 USA). Plasma was diluted 1:8 in urine running buffer which has been observed to increase electrophoretic separation of globulin proteins comprising most blood proteins in elasmobranchs (14). Very poor fraction resolution was observed at a working dilution of 1:2 (Supplementary Figure 1D). There were no significant differences in resolution or fractions with dilutions of 1:4 through 1:8. Fraction delimits were placed based on conventions applied from previous elasmobranch publications (see discussion) while honoring inflection points as well as consistently observed fractions and were completed by one author (J.S.). Absolute protein (g/dL) values for each fraction were obtained by multiplying the percentages by the total plasma protein. The analyzer was run per manufacturer instructions. The mean (of 3 samples run 8 times) intraassay coefficient of variation for the fractions 1 to 7 were as follows: 24.3, 20.3, 1.6, 8.2, 3.3, 3.1, and 8.0%.

### 2.5 Statistical analysis

Descriptive statistics were obtained through the use of Prism 6.07 (GraphPad Software, Inc., Boston, MA, 02110 USA). The D’Agostino-Pearson test was used to check for normality of the data distribution. The comparison of the CZE data by sample population (aquarium-based vs. free-ranging) and by age/sex was completed using the Mann Whitney test as the data was non normal in distribution. The method comparison analysis was conducted with Spearman’s correlations, Passing Bablok regression, and Bland Altman plots using percentage of fractions (15). Note that these analyses were based on data obtained from samples of 29 free-ranging sharks and 21 aquarium-based sharks due to sample volume limitations. Reference intervals were calculated using MedCalc Statistical Software version 23.1.7 (MedCalc Software Ltd., 8400 Ostend, Belgium). Based on the sample size, calculations were completed using ASVCP guidelines using the robust method and 90% confidence limits (CI) on Box-Cox transformed data (16). Outliers were detected using Tukey’s Test; per ASVCP guidelines, no outliers were removed. Reference intervals were calculated using 62 samples from free-ranging sharks and 23 samples from aquarium-based sharks.

## 3 Results

**Figure 1:**
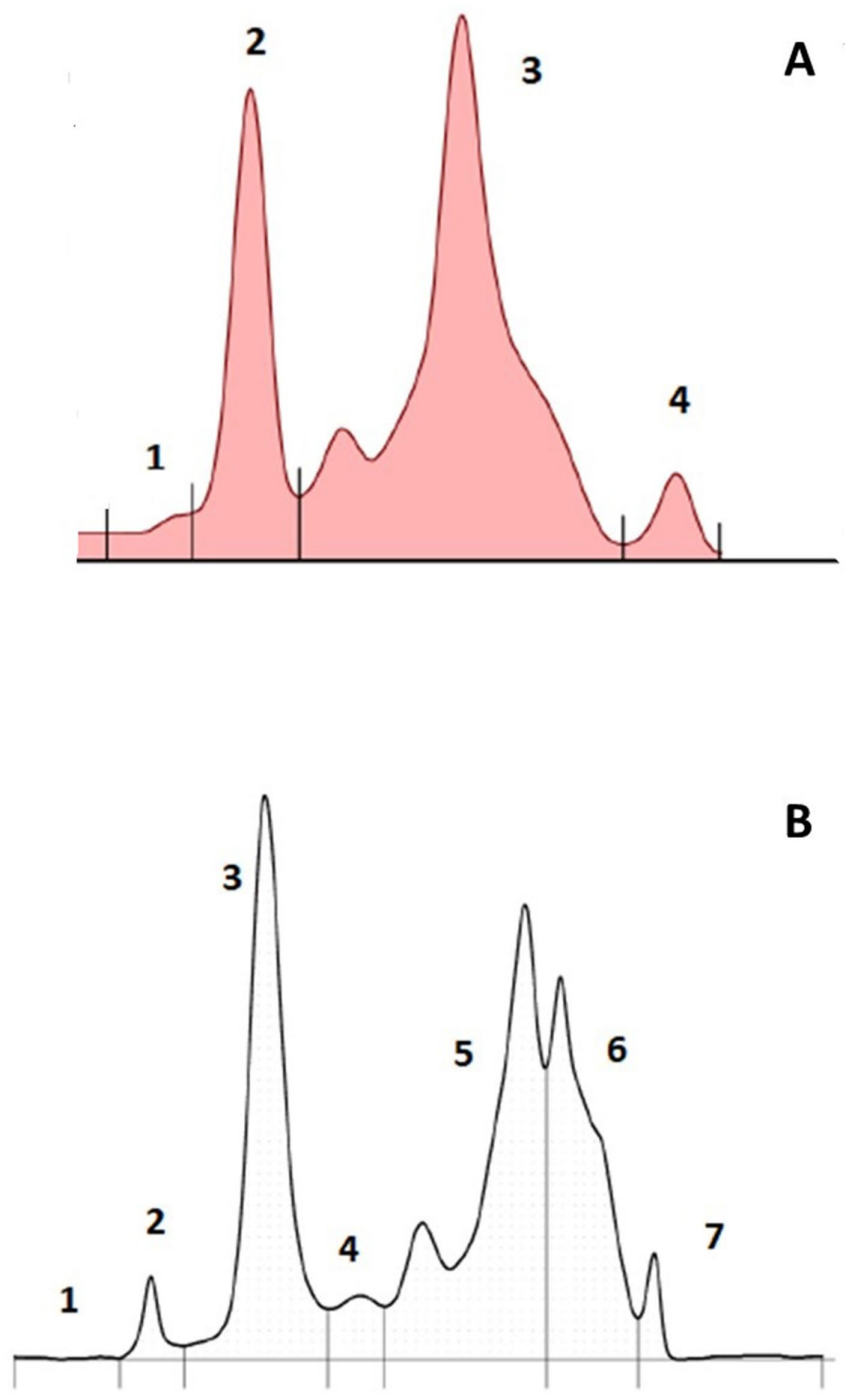
Representative paired plasma AGE **(A)** and CZE **(B)** electrophoretograms from an adult male free-ranging sand tiger shark (*Carcharias taurus*).

The AGE method resolved 4 fractions (Figure 1A). While a minor band could be found migrating anodic to the major band in fraction 3, it was not consistently observed. Based on fraction delimitation methods used previously for other elasmobranch species, it was placed in fraction 3. The CZE method resolved 7 fractions (Figure 1B). Similarly, fraction 5 had a diminutive subfraction that migrated anodic to the main fraction in some samples, so it was quantified within fraction 5 for data analysis.

**Figure 2:**
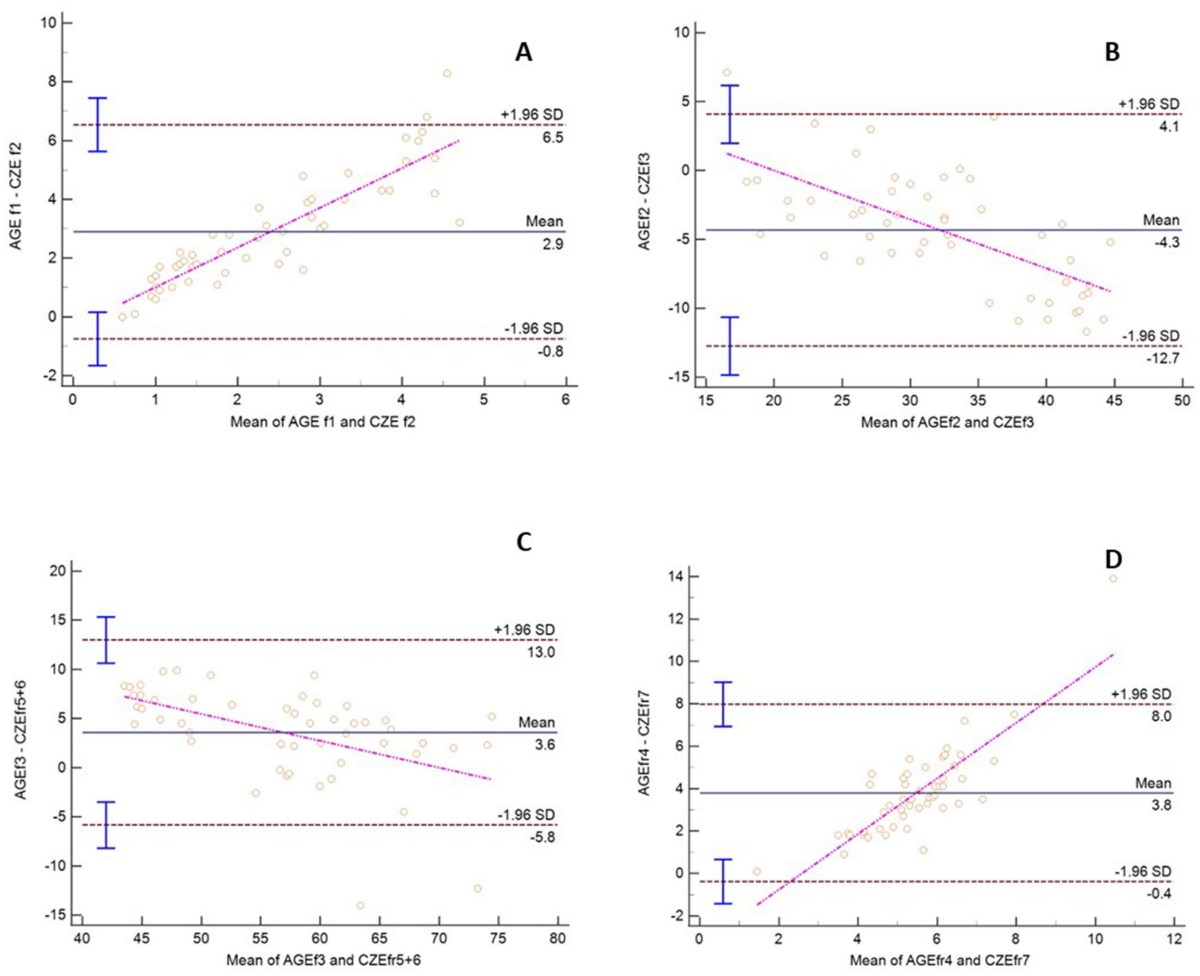
Bland Altman plot comparing AGE and CZE methods for sand tiger shark (*Carcharias taurus*) plasma protein fractions. The dashed horizontal lines represent the 95% limits of agreement, and the center horizontal solid line represents the mean difference between the methods. The pink line is the line of regression. The y-axis is the difference between the similar fractions by method and the x-axis is the mean of values for the same fractions.

Method comparison statistical analysis was undertaken by grouping the protein fractions from each method based on their migration and appearance. Strong to very strong significant correlations were observed between these fractions by Spearman’s correlation (Table 1). By Passing Bablok regression and Bland Altman analysis, both constant and proportional errors were observed as well as a bias between the methods (Figure 2). Specific to the latter point, results from AGE overestimated 3 of the 4 fractions analyzed versus CZE (Table 1).

**Table 1:** Comparison of relative percentage of sand tiger shark (*Carcharias taurus*) plasma (*n* = 50) fractions between AGE and CZE methods using Spearman’s correlation, Passing-Bablok regression, and Bland Altman plot.

Fraction comparison	AGE Median (IQR)^a^	CZE Median (IQR)^a^	Spearman’s correlation (*p*-value)	Passing Bablok y-intercept (95% CI)^a^	Passing Bablok Slope (95% CI)^a^	Bland Altman Mean bias (SD)^a^
AGE 1 vs. CZE 2	3.50 (2.40–4.60)	0.85 (0.60–1.10)	0.57 (*p* < 0.0001)	−1.22 (−3.75–0.28)	5.60 (3.71–9.00)	2.90 (1.86)
AGE 2 vs. CZE 3	30.35 (27.70–33.80)	33.65 (30.20–36.60)	0.92 (p < 0.0001)	4.83 (2.19–7.98)	0.71 (0.63–0.80)	−4.34 (4.29)
AGE 3 vs. CZE 5 + 6	58.95 (55.50–62.20)	56.55 (49.40–59.10)	0.89 (*p* < 0.0001)	14.88 (10.58–19.52)	0.82 (0.72–0.89)	3.57 (4.81)
AGE 4 vs. CZE 7	7.40 (6.60–8.00)	3.60 (3.30–3.90)	0.42 (*p* = 0.0023)	−6.20 (−15.7--2.12)	3.67 (2.53–6.33)	3.80 (2.13)

^aIQR, interquartile range; CI, confidence interval; SD, standard deviation.^

Minor but significant differences were found for some of the CZE protein fractions when comparing aquarium-based to free-ranging sand tiger sharks (Table 2). Adult free-ranging sharks had significantly less fraction 3 and more fraction 6 versus free-ranging juvenile sharks (Table 3). Female adult free-ranging sharks exhibited lower levels of fraction 3 and 5 versus male adult free-ranging sharks (Table 4). Similar comparisons of juvenile sharks were not conducted due to the low number of male samples. Overall reference intervals using the robust method are presented in Tables 5, 6.

**Table 2:** Blood plasma protein concentration (total and fractions from CZE) for aquarium-based (*n* = 23) and free-ranging (*n* = 62) sand tiger sharks (*Carcharias taurus*).

Measurand	Aquarium-based	Free-ranging	*p* value
Total protein	3.4 (3.1–4.4)	3.2 (3.1–3.4)	0.12
CZE Fraction 1	0.01 (0.01–0.01)	0.01 (0.01–0.01)	0.92
CZE Fraction 2	0.02 (0.01–0.02)	0.03 (0.03–0.04)	0.0002
CZE Fraction 3	1.18 (1.03–1.43)	1.10 (0.96–1.23)	0.45
CZE Fraction 4	0.19 (0.17–0.22)	0.17 (0.16–0.19)	0.04
CZE Fraction 5	1.03 (0.97–1.16)	0.95 (0.87–1.01)	0.06
CZE Fraction 6	0.90 (0.69–1.08)	0.80 (0.65–0.98)	0.29
CZE Fraction 7	0.13 (0.10–0.17)	0.12 (0.11–0.13)	0.28

Data were non-normal in distribution: median (interquartile range) and Mann–Whitney *p*-value are presented. All values are g/dL.

**Table 3:** Blood plasma protein concentration (total and fractions from CZE) for adult (*n* = 35) and juvenile (*n* = 24) free-ranging sand tiger sharks (*Carcharias taurus*).

Measurand	Adult	Juvenile	*p* value
Total protein	3.3 (2.8–3.9)	3.2 (2.8–3.4)	0.17
CZE Fraction 1	0.01 (0.01–0.01)	0.01 (0.01–0.01)	0.77
CZE Fraction 2	0.03 (0.02–0.04)	0.04 (0.03–0.05)	0.009
CZE Fraction 3	0.93 (0.83–1.07)	1.36 (1.23–1.52)	<0.0001
CZE Fraction 4	0.17 (0.14–0.20)	0.16 (0.16–0.19)	0.77
CZE Fraction 5	0.99 (0.84–1.22)	0.94 (0.82–1.01)	0.22
CZE Fraction 6	1.08 (0.80–1.33)	0.42 (0.31–0.54)	<0.0001
CZE Fraction 7	0.12 (0.10–0.14)	0.12 (0.11–0.14)	0.56

Data were non-normal in distribution: median (interquartile range) and Mann–Whitney *p*-value are presented. All values are g/dL.

**Table 4:** Blood plasma protein concentration (total and fractions from CZE) for female (*n* = 22) and male (*n* = 13) adult free-ranging sand tiger sharks (*Carcharias taurus*).

Measurand	Female	Male	*p* value
Total protein	3.1 (2.8–3.5)	3.8 (3.3–4.1)	<0.0001
CZE Fraction 1	0.01 (0.01–0.01)	0.01 (0.01–0.01)	0.95
CZE Fraction 2	0.03 (0.02–0.04)	0.03 (0.02–0.04)	0.24
CZE Fraction 3	0.91 (0.78–0.97)	1.01 (0.94–1.22)	0.0028
CZE Fraction 4	0.16 (0.13–0.19)	0.19 (0.17–0.21)	0.04
CZE Fraction 5	0.88 (0.74–1.09)	1.17 (0.96–1.34)	0.01
CZE Fraction 6	1.00 (0.80–1.28)	1.19 (0.75–1.36)	0.49
CZE Fraction 7	0.11 (0.10–0.14)	0.12 (0.10–0.14)	0.63

Data were non-normal in distribution: median (interquartile range) and Mann–Whitney *p*-value are presented. All values are g/dL.

**Table 5:** Capillary zone electrophoresis (CZE)-based reference intervals from free-ranging sand tiger sharks (*Carcharias taurus*) (*n* = 62).

Measurand	# Outliers	Mean	SD^a^	Median	Min	Max	*p*-value^b^	Dist^a^	LRL of RI^a^	URL of RI^a^	LRL CI^a^	URL CI^a^
Total protein	0	3.3	0.5	3.2	2.5	4.4	0.08	G^a^	2.2	4.2	2.0-2.4	4.0–4.4
Fraction 1	9 (0×2, 0.2×6, 0.5)	0.01	0.006	0.01	0	0.05	<0.0001	NG^a^	0.001	0.023	0-0.001	0.021–0.025
Fraction 2	2 (0.08, 0.1)	0.04	0.02	0.03	0.01	0.10	<0.0001	NG	0.0	0.07	0–0	0.06–0.08
Fraction 3	1 (2.53)	1.13	0.32	1.10	0.40	2.53	<0.0001	NG	0.46	1.76	0.28–0.64	1.58–1.93
Fraction 4	2 (0.25, 0.3)	0.17	0.04	0.17	0.11	0.30	0.0092	NG	0.10	0.24	0.08–0.11	0.23–0.36
Fraction 5	1 (1.66)	0.98	0.22	0.95	0.66	1.66	0.02	NG	0.51	1.39	0.42–0.59	1.29–1.49
Fraction 6	0	0.82	0.38	0.80	0.15	1.70	0.03	NG	0.04	1.59	0–0.16	1.46–1.72
Fraction 7	0	0.12	0.03	0.12	0.07	0.18	0.35	G^a^	0.06	0.17	0.05–0.07	0.16–0.18

Values are g/dL. The data was transformed and analyzed using the robust method. No outliers were removed.

^aCI, confidence interval; Dist, data distribution; G, Gaussian distribution; LRL, lower reference limit; NG, Non Gaussian distribution; RI, reference interval; SD, standard deviation; URL, upper reference limit.^

^bp value from the D’Agostino-Pearson test for normality.^

**Table 6:** Capillary zone electrophoresis (CZE)-based reference intervals from aquarium-based sand tiger sharks (*Carcharias taurus*) (*n* = 23).

Measurand	# Outliers	Mean	SD^a^	Median	Min	Max	*p*-value^b^	Dist^a^	LRL of RI^a^	URL of RI^a^	LRL CI^a^	URL CI^a^
Total protein	0	3.6	0.8	3.4	2.2	4.9	0.22	G^a^	1.8	5.3	1.3-2.1	4.6–5.8
Fraction 1	4 (0, 0.02, 0.02, 0.02)	0.01	0.005	0.01	0.0	0.02	0.02	NG^a^	0.0	0.019	0-0	0.017–0.022
Fraction 2	2 (0.06, 0.08)	0.02	0.018	0.02	0.01	0.08	<0.0001	NG	0.0	0.06	0–0	0.04–0.07
Fraction 3	0	1.18	0.30	1.18	0.70	1.62	0.14	G	0.55	1.82	0.40–0.72	1.64–1.97
Fraction 4	1 (0.39)	0.20	0.06	0.19	0.11	0.39	0.01	NG	0.05	0.33	0.01–0.10	0.28–0.38
Fraction 5	5 (0.6, 0.63, 1.57, 1.67, 1.78)	1.10	0.30	1.03	0.60	1.78	0.28	G	0.37	1.68	0.24–0.59	1.46–1.92
Fraction 6	0	0.93	0.34	0.90	0.40	1.67	0.33	G	0.16	1.61	0–0.35	1.36–1.87
Fraction 7	0	0.13	0.04	0.13	0.07	0.20	0.02	NG	0.04	0.23	0.01–0.07	0.20–0.25

Values are g/dL. The data was transformed and analyzed using the robust method. No outliers were removed.

^aCI, confidence interval; Dist, distribution; G, Gaussian distribution; LRL, lower reference limit; NG, Non Gaussian distribution; RI, reference interval; SD, standard deviation; URL, upper reference limit.^

^bp value from the D’Agostino-Pearson test for normality.^

## 4 Discussion

Several similarities were observed in the sand tiger shark electrophoretograms by AGE and CZE methodologies. A visible peak for an albumin migrating fraction (CZE fraction 2) was resolved which was not readily apparent by AGE fraction 1. Notably, both of these CZE and AGE fractions composed less than 3.5% of the total protein. To this observation, elasmobranch plasma does not contain albumin and, instead, this small fraction seen by CZE has been assigned to represent high density lipoprotein (HDL) (9, 17). Fraction 2 (AGE) showed the same migration characteristics as CZE fraction 3 and had a very strong significant correlation. In addition, AGE fraction 3 was similar in migration to CZE fractions 5 + 6 with also a strong significant correlation. If likened to the EPH of the cownose ray, it would be proposed that CZE fraction 3 and CZE fractions 5 + 6 represent low density lipoprotein (LDL) and very low density lipoprotein (VLDL), respectively (9). However, without additional studies by cholesterol electrophoresis or other more advanced methods, these lipoprotein fraction assignments are speculative.

Notably, CZE consistently resolved 7 fractions including a new fraction previously unresolved (fraction 4) and the appearance of fraction 6 which likely was present in the beta migrating fraction of the AGE method (e.g., AGE fraction 3). The increased resolution is consistent with that previously reported in other non-mammalian species (8). Of the fraction comparisons, it was notable that AGE methods resulted in a significantly higher gamma globulin migrating fraction (AGE fraction 4, CZE fraction 7). Overall, 3 of the 4 AGE fractions were higher than the corresponding CZE fractions. Differences in resolution as well as fraction values between the two methods have been previously reported in avian and reptile species as well as dogs, cats, and dolphins (18–21). This may be reflective of differences in the use of protein staining and gel scanning (AGE) versus ultraviolet protein detection methods (CZE). In addition, there may be fraction migration differences which are method related. In mammals, UV detection methods have been reported to be more accurate than staining (22). Given the high concentrations of lipoproteins in elasmobranch samples, this may also be a major factor both in fraction migration and staining. Overall, together with the definition of both proportional and constant error between the two methods, this reinforces the concept that method-specific RI should always be used in the interpretation of EPH for all species.

The sand tiger shark AGE electrophoretogram is similar to that observed in the nurse, blacknose, blacktip, lemon, bull, sandbar, tiger and bonnethead sharks (10, 11, 13). Additionally, previously published electrophoretic data on the Atlantic sharpnose and spiny dogfish sharks, despite the absence of corresponding electrophoretograms, indicate a similar fraction pattern, with two prominent protein fractions (AGE 2 and 3) although fraction numerical assignments/names differ among the publications (5). The low concentration or relative abundance of AGE fraction 1 was also observed in lemon and sandbar sharks while the gamma globulin fraction (AGE fraction 4) is very similar across all studies (5, 10–13). The sand tiger shark CZE electrophoretogram differs from that reported in the nursehound shark (14). While fraction 2 is clearly present in both methods, the other mid globulin migrating fractions are resolved differently. This may be related to differences in methods between the two studies including sample dilution and CZE equipment. The CZE gamma globulin migrating fraction (fraction 7) was similar in quantitation to that of the nursehound shark. Additional studies should be undertaken to address these method and fraction assignment differences in AGE and CZE to aim for standardization among veterinary reference laboratories and research studies of elasmobranch species.

A few minor, yet significant, differences were observed in the CZE fractions of the aquarium-based versus free-ranging sand tiger sharks. This information should be considered preliminary given the smaller sample size of the aquarium-based sharks in the current study. Free-ranging male sand tiger sharks have significantly higher levels of CZE fraction 3 and 5 versus female sharks. Increases in the relative abundance of fraction 3 for aquarium versus free-ranging sharks was also reported for CZE fraction 4 in nursehound shark plasma samples (14). The increase in the relative abundance of CZE fraction 5 is contrary to that reported in female bamboo sharks where this AGE defined beta migrating fraction was found to be higher versus males (12). These types of changes may be related to differences in lipoprotein concentrations as well as other acute phase reactants and proteins which may migrate in these fractions. Adult free-ranging sharks were found to have lower CZE fraction 3 and significantly higher CZE fraction 6 than juvenile free-ranging sharks. The increase in CZE fraction 6 is similar to that reported in aquarium based nursehound shark using CZE methods. Notably, seasonal, life stage, and sex differences prompted variation in several measures including cholesterol, triglycerides, and free fatty acids in free-ranging nurse sharks (23). In addition, using AGE as well as other biochemistry analyses, several differences in plasma biochemistry were reported between juvenile and adult free-ranging sand tiger sharks (4). In total, these findings may reflect differences in sampling periods, sample size, species, and/or methods.

A possible limitation of this study was that samples were frozen prior to analysis. The impact of one freeze–thaw cycle on CZE results in sea turtles has been reported to not be significant but a similar study has not been undertaken in elasmobranchs (18). Of note, the sample storage conditions in the current study were the same for both methods and that used in the nursehound shark study (14). Different sampling and storage protocols were also present in the current study given the use of samples from different aquaria and free-ranging shark studies. Specifically, some free-ranging shark samples were stored temporarily at −20°C in the field laboratory before transferring to -80°C for storage whereas samples from aquarium-based sharks were stored at −80°C. There was also variation in the total storage time prior to analysis. While no specific stability tests were performed in the current study, limited testing by the authors (J.S. and C.C.) indicate that samples appear stable at −20°C over at least a short term and a single freeze–thaw cycle is also acceptable. As the use of CZE in elasmobranch species expands for both research and clinical applications, future studies should fully address storage effects over time as well as freeze–thaw effects. The possibility of plasma differences due to season as well as differing husbandry and environmental conditions among the aquariums were also not considered. Also, while the general health of the sharks in the aquaria was monitored before and after sample acquisition, the presence of subclinical inflammation or disease in this group as well as the free-ranging group cannot be ruled out.

It is also important to note that data from CZE and AGE methods are derived from a combination of these previous elasmobranch publications and extensive experience with the protein electrophoresis of non-mammalian species (J.S. and C.C.). With the goals of placing consistent fraction delimits, inflection points are chosen but there is an element of subjective assessment which is understood as a limitation of electrophoresis methods (8). In addition, in the present study, a small area for fraction 1 was recognized in CZE. While a visible peak was not present in all sand tiger shark samples, it has been observed in other elasmobranch species (J.S and C.C., personal observation). It is acknowledged that the quantitation of minor peaks like CZE 1 and 2 and AGE 1 is done so at the expense of a higher coefficient of variation. Future studies should better address changes in these types of fractions as related to health status, season, sex, and husbandry to understand their utility in research and clinical applications. Overall, it is important in studies such as the current one to include representative electrophoretograms for other laboratories to review and utilize.

The application of the newly established sand tiger shark CZE reference intervals requires further review for applicability as a health assessment tool in aquaria and free-ranging sharks. Further studies should include comparison to other routine bloodwork including complete blood counts in healthy sharks and those with defined disease states in addition to the validation of specific acute phase protein reagents (10). Given the variations in electrophoretograms by species and method, the reference intervals can be considered a foundation for other studies using these methods in elasmobranch species.

## Data Availability

The raw data supporting the conclusions of this article will be made available by the authors, without undue reservation.
