# Supplementary figures and images for: Capillary zone and agarose plasma protein electrophoresis in the sand tiger shark (Carcharias taurus)
Source: Front Vet Sci. 2025 May 9;12:1580744. doi: 10.3389/fvets.2025.1580744 (PMC12100930; doi:10.3389/fvets.2025.1580744)

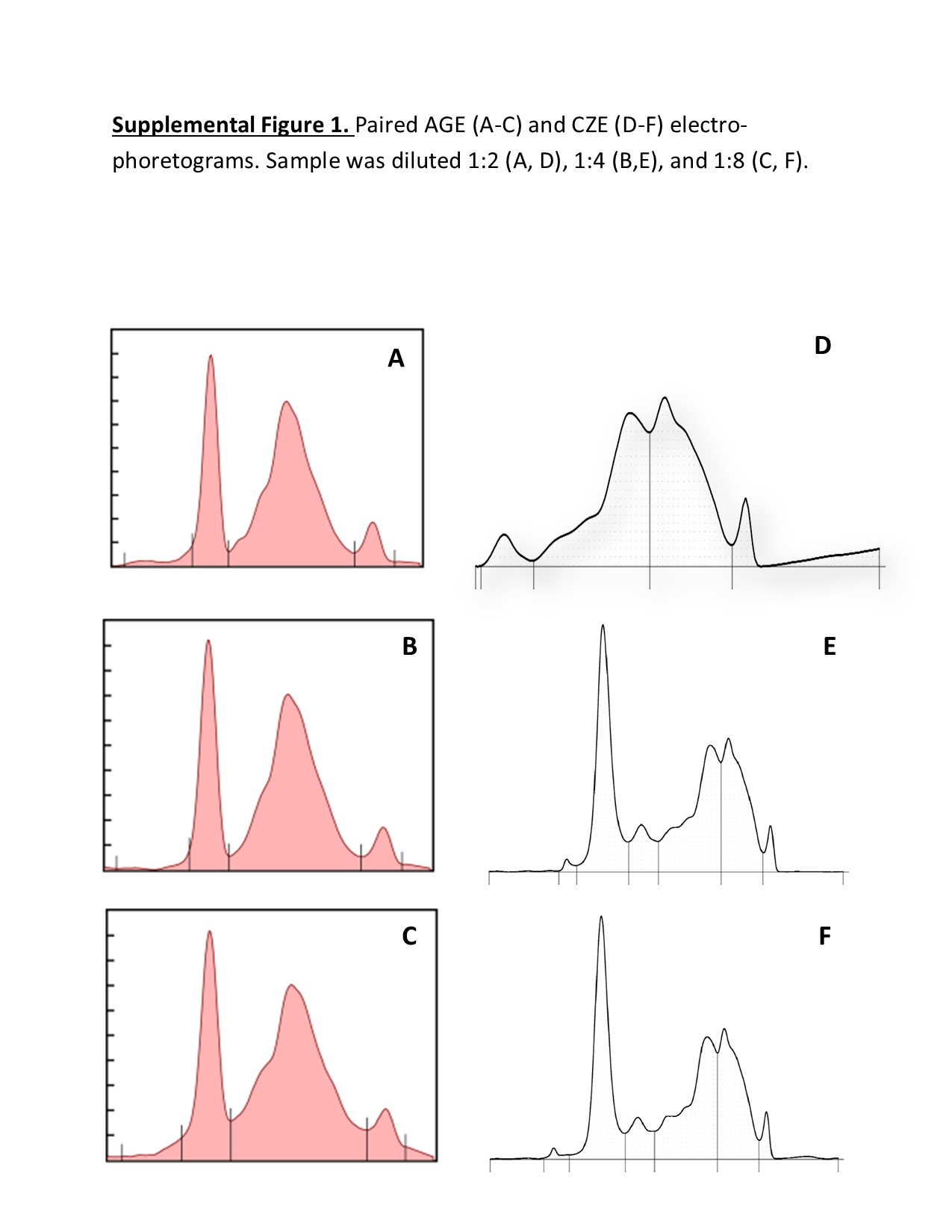

Supplement: Supplementary file 1 [file Image_1.jpeg]
